# Prison for Boys—and the Remedy

**Published:** 1917-12-22

**Authors:** Henry Bentinck, George Toulmin, Albert Spicer, Louise Oliver, Franca Buxton, Henrietta O. Barnett

**Affiliations:** Chairman. State Children's Association, 53 Victoria Street, S.W. 1.; Vice-Chairmen. State Children's Association, 53 Victoria Street, S.W. 1.; Vice-Chairmen. State Children's Association, 53 Victoria Street, S.W. 1.; Chairman of Parliamentary Committee. State Children's Association, 53 Victoria Street, S.W. 1.; Hon. Treasurers. State Children's Association, 53 Victoria Street, S.W. 1.; Hon. Treasurers. State Children's Association, 53 Victoria Street, S.W. 1.; Hon. Secretary. State Children's Association, 53 Victoria Street, S.W. 1.


*To the Editor of* The Hospital.

### Prison for Boys—and the Remedy.

*To the Editor of* The Hospital.

Sir,—The State Children's Association is deeply concerned at the number of boys between fifteen and eighteen who are being sent to prison for periods varying from seven days to six months. The overcrowded state of reformatories and Borstal institutions—due to the tide of lawlessness which has risen amongst the young as a result of war conditions—is perhaps responsible in some measure for this state of things. Whatever its cause, it is deplorable that young persons should become familiarised with prison life and conditions, and thus be thrust further into crime. For our prison system—as we know to our cost—is never reformative. Moreover, imprisonment is unnecessary, for the Justices have another method which they can employ for young delinquents, whose desire for adventure and whose inexperience of life have landed them in the Juvenile or in the ordinary Police Court. In some London and provincial Courts the system of probation is used with such admirable effect that numbers of young persons, after a probationary term, make no further appearance before the Justices. In others this method is employed but little, and in a fashion which prohibits success.

In August last the Home Office issued a valuable letter to Justices, calling their attention to the need for an increased use of probation, and pointing out the advisability of securing voluntary helpers, to prevent probation officers being overburdened with cases, as some of them undoubtedly are.

The purpose of this letter, therefore, is to appeal to such of your readers—men or women—as have sympathy with and understanding of the young, to offer their services to their local Bench of Magistrates as voluntary probation officers for one, two, or more children, as their time and powers permit. We venture to affirm with confidence that under the influence of a steadying friendship the greater number of the juvenile offenders of to-day would become trustworthy citizens of to-morrow.—We are, yours truly,

(Signed) Lytton, Chairman.
Henry Bentinck, George Toulmin, } Vice-Chairmen.
Albert Spicer, Chairman of Parliamentary Committee.
Louise Oliver, Franca Buxton, } Hon. Treasurers.
Henrietta O. Barnett, Hon. Secretary.

State Children's Association,
53 Victoria Street, S.W. 1.
December 1, 1917.

[In our Notes this week a third alternative is suggested.—Ed. The Hospital.]

